# Memory, mood and associated neuroanatomy in individuals with steroid sulphatase deficiency (X‐linked ichthyosis)

**DOI:** 10.1111/gbb.12893

**Published:** 2024-05-05

**Authors:** Georgina H. Wren, Jessica Flanagan, Jack F. G. Underwood, Andrew R. Thompson, Trevor Humby, William Davies

**Affiliations:** ^1^ School of Psychology Cardiff University Cardiff UK; ^2^ Division of Psychological Medicine and Clinical Neurosciences and Centre for Neuropsychiatric Genetics and Genomics School of Medicine, Cardiff University Cardiff UK; ^3^ Neuroscience and Mental Health Innovation Institute Cardiff University Cardiff UK; ^4^ South Wales Clinical Psychology Doctoral Programme Cardiff and Vale University Health Board Cardiff UK

**Keywords:** dehydroepiandrosterone sulphate, globus pallidus, hippocampus, online survey, Xp22.31

## Abstract

Steroid sulphatase (STS) cleaves sulphate groups from steroid hormones, and steroid (sulphate) levels correlate with mood and age‐related cognitive decline. In animals, STS inhibition or deletion of the associated gene, enhances memory/neuroprotection and alters hippocampal neurochemistry. Little is known about the consequences of constitutive STS deficiency on memory‐related processes in humans. We investigated self‐reported memory performance (Multifactorial Memory Questionnaire), word‐picture recall and recent mood (Kessler Psychological Distress Scale, K10) in adult males with STS deficiency diagnosed with the dermatological condition X‐linked ichthyosis (XLI; *n* = 41) and in adult female carriers of XLI‐associated genetic variants (*n* = 79); we compared results to those obtained from matched control subjects [diagnosed with ichthyosis vulgaris (IV, *n* = 98) or recruited from the general population (*n* = 250)]. Using the UK Biobank, we compared mood/memory‐related neuroanatomy in carriers of genetic deletions encompassing *STS* (*n* = 28) and non‐carriers (*n* = 34,522). We found poorer word‐picture recall and lower perceived memory abilities in males with XLI and female carriers compared with control groups. XLI‐associated variant carriers and individuals with IV reported more adverse mood symptoms, reduced memory contentment and greater use of memory aids, compared with general population controls. Mood and memory findings appeared largely independent. Neuroanatomical analysis only indicated a nominally‐significantly larger molecular layer in the right hippocampal body of deletion carriers relative to non‐carriers. In humans, constitutive STS deficiency appears associated with mood‐independent impairments in memory but not with large effects on underlying brain structure; the mediating psychobiological mechanisms might be explored further in individuals with XLI and in new mammalian models lacking STS developmentally.

## Graphical abstract

Animals in which the steroid sulphatase protein is absent or non‐functional show evidence for enhanced memory and longevity. Here we show that, in contrast, humans carrying deletions of the *STS* gene show poorer memory performance (and more adverse mood symptoms) than non‐carriers, but no differences from controls regarding associated neuroanatomy.

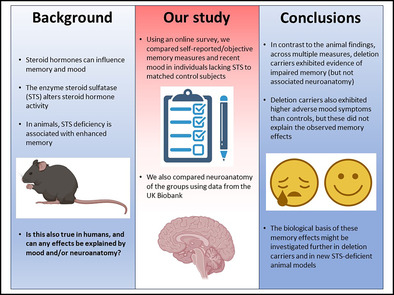

## 1 INTRODUCTION

The steroid sulphatase (STS) enzyme cleaves sulphate groups from a variety of steroid hormones (e.g., dehydroepiandrosterone sulphate, DHEAS), thereby altering their solubility and/or activity.^1^ There is some evidence from animal models for an association between STS activity, memory, hippocampal neurochemistry and neuroprotection. In adult rats, acute pharmacological inhibition of STS reverses scopolamine‐induced amnesia in a passive avoidance test and improves learning and memory performance in the Morris Water Maze^2^, ^3^, ^4^ possibly via initial elevated peripheral DHEAS levels^5^ and/or downstream hippocampal acetylcholine release.^6^ More recently, acute intra‐hippocampal and peripheral infusion of STS inhibitor in rodents was found to reduce spatial learning and memory impairments, hippocampal electrophysiological abnormalities and hippocampal synaptic plasticity changes induced as a consequence of intra‐hippocampal administration of amyloid beta oligomers^7^, ^8^; longer‐term oral administration of the STS inhibitor STX‐64 alleviated memory impairments and hippocampal and cortical neuropathology in a genetic mouse model of relevance to Alzheimer's disease (AD).^7^ Intriguingly, worms lacking the STS orthologue *sul‐2* (or in which sul‐2 was acutely inhibited) exhibited enhanced longevity as well as some degree of protection against AD‐related pathology.^7^ Our previous work in 39,X^Y*^O male mice, in which the *Sts* gene and a small number of adjacent pseudoautosomal genes are deleted as a consequence of an end‐to‐end fusion of the X and Y chromosomes, showed markedly altered hippocampal serotonergic neurochemistry relative to wildtype controls, but no clear differences from controls with respect to performance on hippocampal‐dependent foraging and ‘object‐in‐place’ tasks.^9^, ^10^ 39,X^Y*^O mice also show substantially altered hippocampal expression of *C1qc*,^11^ a gene encoding the C‐chain of the C1q complex implicated in synaptic pruning during neurodevelopment, ageing and neurodegeneration.^12^, ^13^

In humans, deletion copy number variants (CNVs) at Xp22.31 encompassing *STS* (present in approximately 1 in 1500 males^14^), or non‐synonymous single nucleotide variants within the gene, are associated with the rare dermatological condition X‐linked ichthyosis (XLI), as well as an increased predisposition to neurodevelopmental conditions and adverse mood symptoms; XLI is not typically associated with significant effects on IQ in man, or learning deficits in STS‐deficient animal models.^15^, ^16^ *STS* is expressed throughout the developing and adult human brain, with highest early expression in the basal ganglia (https://www.gtexportal.org/home/GTex).^17^ An initial analysis of around 20,000 individuals from the UK Biobank neuroimaging sample indicated a significantly smaller volume of basal ganglia subregions in deletion carriers that might partially explain the association with neurodevelopmental/mood conditions, but this preliminary analysis did not implicate gross volumetric differences between deletion carriers and non‐carriers with respect to other subcortical structures including the hippocampus.^18^ In terms of general cognition, Xp22.31 deletion carriers in the UK Biobank have been reported to exhibit a mild impairment on a measure of Fluid Intelligence, but do not differ significantly from non‐carriers with respect to academic achievement as indexed by highest level of academic qualification obtained.^18^ To date, there has been little work on specific aspects of cognition in individuals with XLI or Xp22.31 deletion carriers, or their relationship with mood symptoms. In the UK Biobank sample, deletion carriers tended to perform more poorly than non‐carrier participants across tasks indexing general executive function, processing speed, reaction time, short‐term memory and attention.^18^

Overall, these cross‐species data, when considered in combination with findings indicating a relationship between circulating/brain DHEA(S) levels and age‐related cognitive decline and neurodegeneration in humans,^19^, ^20^ suggest the possibility that constitutive STS deficiency might impact upon aspects of memory, on neurodegenerative processes, and upon the structure/function of the hippocampus (and connected brain regions). The nature of any relationships may be modified by mood: elevated depressive traits are linked to memory disruption^21^ and systemic DHEA(S) levels influence mood.^22^

Here, we first compared self‐reported memory performance, word‐picture recall and mood in male and female carriers of XLI‐associated genetic variants to that of sex‐matched general population controls using an online survey; we also included an additional control group of males and females with ichthyosis vulgaris (a dermatological condition presenting similarly to XLI and with similar effects on mood,^15^ but with no anticipated effects on memory). Prior to the study, it was difficult to predict whether constitutive STS deficiency would be associated with enhanced memory (as suggested by the animal model data) or with impaired memory (as suggested by limited pre‐existing human data). Second, we compared mood and memory‐associated neuroanatomy (hippocampal subregions and associated cortical structures and white matter tracts) in detail in Xp22.31 deletion carriers and non‐carriers using the latest UK Biobank neuroimaging sample of approximately 40,000 individuals; cortical morphology and white matter tract integrity have not been examined in Xp22.31 deletion carriers previously. This analysis was exploratory and we had no specific a priori hypotheses about affected regions, direction of effect or effect size. Finally, we sought to confirm initial basal ganglia structure findings in deletion carriers in the extended UK Biobank dataset.

## 2 MATERIALS AND METHODS

### 2.1 Online survey

#### 2.1.1 Ethical permissions

Conduct of the survey was approved by Cardiff University School of Psychology Ethics Committee (approval numbers EC.22.04.26.6565 and EC.22.10.11.6642) and subjects provided informed consent.

#### 2.1.2 Participants

Adult participants with a clinical diagnosis of X‐linked ichthyosis (*n* = 41 males) or ichthyosis vulgaris (*n* = 30 males, *n* = 68 females), or confirmed carriers of XLI‐associated genetic variants (*n* = 79 females) were recruited based upon past contact, via charities, or via social media including patient support groups. Age‐matched male (*n* = 123) and female (*n* = 127) participants from the United Kingdom or United States general population were recruited via Prolific (https://www.prolific.co/) and were reimbursed in accordance with their policies.

#### 2.1.3 Survey structure

The survey was generated using Qualtrics software (https://www.qualtrics.com) and participants were supplied with the URL. Initially, participants were shown a list of 15 words for 30 s and a montage of 20 pictures for 45 s and instructed to remember them without the use of memory aids. A number of demographic measures were collected including participant age, country of residence, sex at birth and highest level of education (up to high school, greater than high school). Participants were then asked whether they had a parent, grandparent or sibling who had been diagnosed with a medical condition affecting memory (e.g., dementia) and, if so, to specify which relative; they were also asked about how their skin condition (if present) was diagnosed and to rate its average severity across life (based upon Congenital Ichthyoses Severity Index,^23^ scale range 2–8). Participants next completed the Kessler Psychological Distress Scale (K10), a 10‐item 5 point response questionnaire assessing recent depression/anxiety‐related traits (possible score range 10–50, with a score of ≥20 being consistent with significant psychological distress).^24^ Following this, participants completed the Multifactorial Memory Questionnaire (MMQ), a tripartite questionnaire assessing recent contentment with memory performance (18 items each scored 0–4, higher scores indicating greater contentment), perceived memory ability (20 items each scored 0–4, higher scores indicating better subjective memory ability) and use of strategies to aid memory (19 items, each scored 0–4 with higher scores indicating greater memory aid usage).^25^ Finally, as an objective measure of current memory performance, participants were asked to identify which words from a randomly‐ordered list of 30 they had been presented with at the start of the survey (maximum 15 choices to guess all 15 correct answers) and which pictures from a randomly ordered montage of 40 they had been presented with at the start of the survey (maximum 20 choices to identify all 20 correct answers). The number of correctly‐identified words was multiplied by four, and the number of correctly‐identified pictures by three, and the totals summed to generate a ‘word‐picture recall index’ with possible scores ranging from 0 to 120. Duration of survey completion was recorded (and <2% of participants excluded where this was >5400 s indicating failure to end the survey) to control for the length of time between the stimulus presentation and recall phases. Participants from the ichthyosis groups were given the opportunity to complete additional neuropsychological tests taxing memory (Forward Digit Span, Backward Digit Span, Digit Symbol Matching, Verbal Paired Associates and Visual Paired Associates) from the online ‘TestMyBrain’ battery (https://testmybrain.org/) and one participant drawn at random was awarded a £50 shopping voucher (ethics approval number EC.22.08.09.6610A); these participants' TestMyBrain data could be cross‐linked to their survey data.

#### 2.1.4 Statistical analysis

Categorical data across groups were compared using chi‐squared or Fisher's exact test, and post hoc comparisons performed using adjusted residuals with Bonferroni correction.^26^ Continuous data were analysed by ANCOVA with factors of GROUP (XLI, IV or general population control) and SEX (male or female) and covariates of age, K10 score and/or highest level of education; our sample size was sufficient to detect a small‐moderate GROUP effect size of *f* >0.18 at 90% power with *α* = 0.05. Significant effects were followed up with post hoc comparisons using Tukey's least significant difference (LSD) test for ANOVA or simple contrasts for ANCOVA. *p*‐Values <0.05 were regarded as being nominally significant with GROUP *p*‐values <0.1 on the memory measures after Benjamini–Hochberg adjustment regarded as surviving correction for multiple comparisons. Correlations were performed using one or two‐tailed Pearson's test or Spearman's test depending upon normality of the data, as determined by Shapiro–Wilk test, and by directionality of the predicted effect.

### 2.2 Neuroanatomical analysis in UK Biobank

#### 2.2.1 Participants and genotyping

Participants were individuals (aged 40–69 years) recruited under UK Biobank informed consent procedures between 2006 and 2010, for which anonymised genotype, cognitive and neuroimaging data were available; males (*n* = 5) and females (*n* = 23) carrying genetic deletions spanning 0.8–2.5 Mb around the *STS* gene were identified as described previously.^18^

#### 2.2.2 Neuroimaging measures

Brain images were acquired using Siemens Skyra 3T scanners in UK Biobank's imaging centres using identical acquisition protocols. T1‐weighted brain images were processed using automated methods implemented in FreeSurfer to obtain grey matter volumetric estimates for the following memory‐associated regions across both hemispheres: hippocampus, hippocampal tail, hippocampal fissure, subiculum head, subiculum body, presubiculum head, presubiculum body, parasubiculum, molecular layer HP head, molecular layer HP body, granule cell‐molecular layer‐dentate gyrus(GC‐ML‐DG)‐head, GC‐ML‐DG‐body, fimbria, HP‐amygdala‐transition‐area (HATA), cornu ammonis (CA) fields—CA1 head, CA1 body, CA3 head, CA3 body, CA4 head, CA4 body and thickness/surface area of global, entorhinal and parahippocampal cortices. Additionally, white matter integrity was assessed by Diffusion Tensor Imaging in the fornix and body and splenium of the corpus callosum through measures of mean diffusivity and fractional anisotropy. In this extended neuroimaging sample, we also attempted to replicate previous findings of lower basal ganglia subregion volume in deletion carriers.^18^

#### 2.2.3 Statistical analysis

Across the overall sample for each neuroimaging measure, outlying values >2.2 times the interquartile range below the first quartile, or above the third quartile, were excluded.^27^ The neuroanatomy of deletion carriers was compared with that of non‐carriers using linear regression controlling for relevant factors (genetic sex, age, intracranial volume, scanning centre and imaging table position). Two‐sided *p*‐values <0.05 were regarded as nominally significant, with *p*‐values <0.1 after Benjamini–Hochberg adjustment regarded as surviving correction for multiple comparisons.

### 2.3 Data availability

Online survey and TestMyBrain data are available within this manuscript or within the Supporting Information file. Neuroimaging and genetic data are available upon application to the UK Biobank resource (https://www.ukbiobank.ac.uk/).

## 3 RESULTS

### 3.1 Online survey

#### 3.1.1 Demographic and clinical variables

The demographic and relevant clinical variables for each group are summarized in Table 1. The mean ages of the six groups were very similar (range 42–49 years), although the IV groups were significantly older than both the XLI (*p* = 0.007) and general population (*p* = 0.015) groups. Although the groups differed in terms of their highest education level, post hoc analysis indicated no significant differences from the null distribution for any group. For all four ichthyosis groups, the majority (>70%) of participants were resident in United Kingdom or United States, so we recruited a general population sample from these countries in an attempt to control for sociocultural and geographical factors. There was a significant difference in the frequency of self‐reported family history of memory disorders across groups, with males with XLI reporting the highest proportion of affected relatives (55%, corrected *p* < 0.05). Male participants with XLI, and male and female participants with IV, did not differ in terms of self‐reported skin severity (moderate in all groups), whilst, as expected, female carriers of XLI‐associated variants exhibited no‐mild skin phenotypes.

**TABLE 1:** Demographic and clinical variables in our online samples. Age and CISI scores are presented as mean values ± standard error of the mean.

Demographic and clinical variables	Sex	Group			Statistical comparison
		XLI	IV	General population	
Age (years)	Male	43.8 ± 2.8	48.6 ± 2.5	44.1 ± 0.6	GROUP: *F*[2,462] = 4.62, *p* = 0.010 SEX: *F*[1,462] = 5.38, *p* = 0.021 GROUP × SEX: *F*[2,462] = 0.17, *p* = 0.84
	Female	41.5 ± 1.1	45.1 ± 1.9	42.1 ± 0.5	
Percentage of sample with highest education level greater than high school	Male	63	73	73	*χ* ^2^[5] = 15.9, *p* = 0.007
	Female	71	88	84	
Percentage of sample with self‐reported positive family history (first or second‐degree relative) for memory‐related condition	Male	55	50	23	*χ* ^2^[5] = 19.0, *p* = 0.002
	Female	39	25	28	
CISI score indexing severity of skin condition	Male	5.1 ± 0.2	4.5 ± 0.2	N/A	GROUP: *F*[1,207] = 3.31, *p* = 0.070 SEX: *F*[1,207] = 35.5, *p* < 0.001 GROUP × SEX: *F*[1,207] = 23.7, *p* < 0.001
	Female	2.9 ± 0.2	4.3 ± 0.2		

#### 3.1.2 Recent mood symptoms (K10 score) and relationship to skin condition

After covarying for age, K10 scores differed significantly by GROUP (*F*[2,458] = 21.3, *p* < 0.001) but not by SEX (*F*[1,458] = 0.013, *p* = 0.91); there was no GROUP × SEX interaction (*F*[2,458] = 2.27, *p* = 0.11; Table 2). Simple contrast analyses suggested that both XLI and IV groups scored significantly higher than general population control groups (*p* < 0.001), but that the former groups were not significantly different from each other (*p* = 0.21). Across the four XLI and IV groups, there was a weak positive correlation between CISI score and K10 score (*r*_s_[208] = 0.12, one‐tailed *p* = 0.045), an effect chiefly driven by an association in female XLI carriers (*r*_s_[72] = 0.25, *p* = 0.016; all other within‐group correlations *r*_s_ ≤0.1, *p* > 0.2).

**TABLE 2:** Mood and memory‐related variables in our online samples. Data are presented as mean values ± standard error of the mean.

Mood and memory variables	Sex	Group			Statistical comparison
		XLI	IV	General population	
K10 score	Male	25.2 ± 1.3	21.3 ± 1.4	19.8 ± 0.7	GROUP: *F*[2,458] = 21.3, *p* < 0.001 SEX: *F*[1,458] = 0.013, *p* = 0.91 GROUP × SEX interaction: *F*[2,458] = 2.27, *p* = 0.11
	Female	24.1 ± 0.7	24.4 ± 1.0	18.9 ± 0.6	
MMQ: contentment with memory	Male	34.7 ± 2.4	39.2 ± 2.7	46.3 ± 1.2	GROUP: *F*[2,456] = 22.0, *p* < 0.001 SEX: *F*[1,456] = 0.031, *p* = 0.87 GROUP × SEX interaction: *F*[2,456] = 0.25, *p* = 0.78
	Female	36.7 ± 1.9	38.7 ± 1.9	46.2 ± 1.2	
MMQ: perceived memory ability	Male	38.1 ± 2.0	44.0 ± 2.3	54.2 ± 1.3	GROUP: *F*[2,454] = 55.1, *p* < 0.001 SEX: *F*[1,454] = 0.42, *p* = 0.52 GROUP × SEX interaction: *F*[2,454] = 0.29, *p* = 0.75
	Female	38.1 ± 1.9	41.2 ± 1.7	54.1 ± 1.2	
MMQ: use of strategies to aid memory	Male	40.8 ± 2.1	36.9 ± 2.2	30.3 ± 1.1	GROUP: *F*[2,444] = 29.0, *p* < 0.001 SEX: *F*[1,444] = 3.32, *p* = 0.07 GROUP × SEX interaction: *F*[2,444] = 0.042, *p* = 0.96
	Female	43.1 ± 1.4	40.2 ± 1.7	32.7 ± 1.1	
Word‐picture recall score	Male	72.6 ± 2.6	84.5 ± 3.2	86.0 ± 1.8	GROUP: *F*[2,431] = 6.88, *p* = 0.001 SEX: *F*[1,431] = 14.8, *p* < 0.001 GROUP × SEX interaction: *F*[2,431] = 1.38, *p* = 0.25
	Female	87.4 ± 2.0	89.7 ± 2.3	94.0 ± 1.7	

#### 3.1.3 Self‐reported memory function

Importantly, the MMQ satisfaction, ability and strategy scores obtained in our general population samples (Table 2) were similar to those previously obtained in age‐matched general population controls.^28^ After covarying for age alone, there was a significant effect of GROUP on ‘contentment with memory’ (*F*[2,456] = 22.0, *p* < 0.001, corrected *p* < 0.1), but no effect of SEX (*F*[1,456] = 0.031, *p* = 0.86), nor any GROUP × SEX interaction (*F*[2,456] = 0.25, *p* = 0.78). Simple contrasts analysis indicated that both XLI and IV groups scored significantly lower than general population groups (*p* < 0.001), but that the former groups did not differ significantly from each other (*p* = 0.10). Covarying for age and K10 score (recent mood), or for age, K10 score and highest level of education, gave rise to an identical pattern of findings. Males with XLI showed a large difference from sex‐matched general population controls (Cohen's *d* = 0.82), whilst the effects in males and females with IV, and in female XLI carriers, compared with sex‐matched general population controls were moderate‐large (Cohen's *d* = 0.51–0.61).

With respect to ‘perceived memory ability’, after covarying for age, there was a significant effect of GROUP (*F*[2,454] = 55.1, *p* < 0.001, corrected *p* < 0.1), but no effect of SEX (*F*[1,454] = 0.42, *p* = 0.52), nor any GROUP × SEX interaction (*F*[2,454] = 0.29, *p* = 0.75). Simple contrasts analysis indicated that both XLI and IV groups scored significantly lower than general population groups (*p* < 0.001), and that the XLI groups scored lower than the IV groups (*p* = 0.031). When adjusting for both age and K10 scores, the pattern of results remained identical apart from the fact that the difference between XLI and IV groups did not quite reach significance (*p* = 0.074); after additional adjustment for highest education level the results did not change further. Males with XLI showed a very large difference from sex‐matched general population controls (Cohen's *d* = 1.22), whilst the effects in males and females with IV, and in female XLI carriers, compared with sex‐matched general population controls were large‐very large (Cohen's *d* = 1.02–1.33).

Following covariation for age alone on the ‘use of strategies to aid memory’ measure, there was a highly significant effect of GROUP (*F*[2,444] = 29.0, *p* < 0.001, corrected *p* < 0.1), no significant effect of SEX (*F*[1,444] = 3.32, *p* = 0.07), and no GROUP × SEX interaction (*F*[2,444] = 0.042, *p* = 0.96). Simple contrasts analysis indicated that both XLI and IV groups scored significantly higher than general population groups (*p* < 0.001), and that the XLI and IV groups scored equivalently (*p* = 0.098). Covarying for age and K10 score, or for three measures (age, K10 score and highest level of education), produced an identical pattern of findings. Males with XLI and female XLI carriers showed similarly large elevated scores compared with sex‐matched general population control groups (Cohen's *d* = 0.84–0.85), whilst both male and female IV groups exhibited moderately higher scores compared with sex‐matched general population control groups (Cohen's *d* = 0.56–0.59).

#### 3.1.4 Word‐picture recall index

Across the whole sample, there was a moderate, highly significant, positive correlation between word recall and picture recall scores (*r*_s_[446] = 0.41, one‐tailed *p* < 0.001), and a small, but highly significant, positive correlation between the composite ‘word‐picture recall score’ and the ‘perceived memory ability’ score from MMQ (*r*_s_[445] = 0.13, one‐tailed *p* = 0.002). Word‐picture recall scores and TestMyBrain (TMB) cognitive scores were available for 10 individuals (two males with XLI, three female carriers of XLI‐associated variants, two males with IV and three females with IV). Despite low power, we found a moderate significant positive correlation between word‐picture recall score and score on the TMB Visual Paired Associates task (*r*_s_[8] = 0.56, one‐tailed *p* = 0.046); there were no other significant correlations (*r*_s_ <0.33, *p* > 0.18). Together, these data indicate that the objectively ascertained memory ability measures, and the self‐reported MMQ measure, are consistent with each other and are likely to index partially overlapping cognitive processes.

Covarying for participants' age and duration of survey completion resulted in a significant effect of GROUP on word‐recall performance (*F*[2,431] = 6.88, *p* = 0.001, corrected *p* < 0.1) and a significant effect of SEX whereby females generally outperformed males (*F*[1,431] = 14.8, *p* < 0.001), but no GROUP × SEX interaction (*F*[2,431] = 1.38, *p* = 0.25). Simple contrast analysis indicated the XLI groups performed significantly worse than both the general population groups (*p* < 0.001, Cohen's *d* = 0.75 and 0.36 for males and females, respectively) and the IV groups (*p* = 0.008), but that there was no significant difference between the IV and general population groups (*p* = 0.86). The pattern of results was identical when K10 score was also included as a covariate.

### 3.2 Neuroanatomical analysis

Following adjustment for relevant covariates, across all of the neuroanatomical measures we assessed, we identified just one nominally‐significant difference between deletion carriers and non‐carriers (Tables S1–S3). Specifically, the volume of the molecular layer of the hippocampal body was greater in deletion carriers than in non‐carriers in the right (but not left) hemisphere (*β* = 0.025, *p* = 0.038); this finding did not survive correction for multiple testing (corrected *p* = 0.85).

Our previous analysis of subcortical structure volumes in UK Biobank participants indicated lower volumes of the right putamen, right globus pallidus and left nucleus accumbens in Xp22.31 deletion carriers relative to non‐carrier controls. In the current extended dataset of approximately 40,000 participants, of these three regions, only the right globus pallidus volume remained significantly smaller in deletion carriers relative to non‐carriers, but only nominally so (*β* = −0.022, *p* = 0.038, corrected *p* = 0.11; Table S4).

## 4 DISCUSSION

Acute or constitutive STS deficiency in animals is associated with enhanced memory and altered hippocampal neurochemistry, but the effects of constitutive STS deficiency in humans on cognition are currently poorly‐understood. In this study, we compared self‐reported and objectively‐determined memory performance, and memory‐associated neuroanatomy, between individuals carrying genetic variants associated with X‐linked ichthyosis and control subjects matched as closely as possible for age, sex and skin condition; we also investigated whether mood might mediate any memory effects. The online survey approach we used maximizes participation by a broad range of individuals and is less subject to biases inherent in ‘in person’ testing.^29^

The results of our ‘word‐picture recall’ test indicate that, in man, genetic variants resulting in the constitutive absence of functional STS protein are associated with lower performance on at least one aspect of memory; as might have been expected, the magnitude of this effect was greater in hemizygous male variant carriers than in heterozygous female carriers. This memory effect is unlikely to be a simple correlate of increased adverse mood symptoms in the carriers of XLI‐associated genetic variants in that word‐picture recall performance in individuals with ichthyosis vulgaris, who exhibit similar levels of skin problems and adverse mood traits, was comparable to that of general population control subjects; moreover, controlling for recent adverse mood traits did not alter the findings. Self‐reported data on ‘perceived memory ability’ across the groups mirrored the pattern of effects from the word‐picture recall test, with greatest perceived impairments in males with X‐linked ichthyosis, and less‐pronounced (but still large) effects in individuals with IV, compared with matched general population controls. Self‐reported data on memory contentment and memory aid usage was further consistent with these findings: individuals carrying XLI‐associated genetic variants reported the lowest contentment/greatest aid usage, and individuals with IV reported lower contentment and greater aid usage than matched general population controls. These data indicate that IV may be associated with perceived memory issues, but not necessarily with objectively indexed memory performance.

Our findings contrast with predictions arising from animal model work. The divergent findings across species may be due to the fact that: (a) there are species differences in cognitive processes; (b) STS inhibition results in incomplete enzyme deficiency (and studies have only examined effects in adulthood) whereas genetic variants such as microdeletions result in complete STS loss throughout life; (c) compounds used as STS inhibitors can have off‐target and oestrogenic effects^30^; and/or (d) XLI‐associated genetic variants in man are typically 1.5–1.7 Mb in size and delete adjacent brain‐expressed genes. Our findings are, however, consistent with previous findings from UK Biobank, where carriers of deletions 0.8–2.5 Mb around the *STS* gene (*n* = 398) exhibited significantly poorer performance on the ‘Pairs Matching’ memory test than non‐carriers (*n* = 97,141).^18^ The consistency of results across differentially‐recruited, genotyped and cognitively tested samples supports the existence of a genuine robust and generalizable effect in Xp22.31 deletion carriers, and addresses study‐specific limitations associated with the two approaches. The effect size in the present study may be larger than that observed in the UK Biobank sample due to: (a) response bias whereby XLI individuals with memory deficits might be more likely to participate (a bias which would also presumably pertain to IV participant recruitment) and/or to (b) the healthy participant bias in the UK Biobank sample, but, overall, a genuine moderate adverse effect on memory in deletion carriers appears plausible. The existence of a transmissible genetic variant (Xp22.31 deletion) which confers worse memory performance could theoretically explain the elevated family history of memory disorders reported amongst males with XLI and carrier females. However, within the XLI male group, of the 16 participants who reported a first‐ or second‐degree relative being affected by a memory disorder, there was no strong evidence for an X‐linked genetic effect specifically, more fathers than mothers were reported to be affected (*n* = 2 vs. *n* = 1), equal numbers of grandfathers and grandmothers were affected (*n* = 6 per group) and similar numbers of brothers and sisters were affected (*n* = 1 and *n* = 0). Potentially, the ‘memory’ effects identified in XLI‐associated genetic variant carriers could be secondary to attentional deficits during learning. We attempted to reduce the attentional demands of the word‐picture recall task through using simple stimuli presented for a reasonable length of time. However, whether memory performance is dissociable from other aspects of cognition (notably IQ, attention, response inhibition) in deletion carriers, or whether the deletion impacts cognition (and academic attainment) more generally might be tested in future work.

Assuming there is a true effect of Xp22.31 deletion on memory which acts largely independently of effects on mood, a key question regards the causal mechanism. The typically sized XLI‐associated deletion covers the *PUDP*, *STS*, *VCX* and *PNPLA4* genes, and the *MIR4767* microRNA.^18^ *PUDP*, *STS* and *PNPLA4* are all expressed in the brain at low‐moderate levels whilst *VCX* expression is testis‐specific and *MIR4767* brain expression is very low (https://www.gtexportal.org/home/). Hence, deficiency for one or more of the former three proteins represents the most plausible biological mechanism; studies investigating cognition in human and animal subjects with null alleles of these individual genes (e.g., mice bearing *Sts*‐specific CRISPR‐generated null alleles) may help to further define causal processes. Theoretically, disrupted sleep, as a consequence of itching or pain, may also be an indirect contributor towards impaired memory performance in individuals with ichthyosis.^31^, ^32^

We used the largest genetic neuroimaging dataset available to test the idea of a neurobiological correlate of memory deficits in individuals with XLI. We found no evidence that Xp22.31 deletion carriers differ substantially from non‐carriers across almost all aspects of gross cortical structure and memory‐associated neuroanatomy; however, given the small sample size of the deletion carrier group, small‐moderate between‐group neuroanatomical differences cannot be definitively ruled out. We did observe greater volume of the molecular layer of the right hippocampal body in deletion carriers; however, this finding did not survive correction for multiple testing and was not also evident in the left hemisphere; it could therefore be a false‐positive result. Nevertheless, the hippocampal molecular layer has been implicated in memory processes and contains Cajal–Retzius cells^33^ in which high STS expression during foetal development and adulthood has been reported.^34^ Moreover, C1Q is highly expressed in microglia and interneurons of this region.^35^ Thus, replication and further investigation of this initial positive neuroimaging finding in deletion carriers and in animal models lacking STS and other Xp22.31 gene orthologues, may be warranted. STS deficiency affects lipid metabolism, notably ceramide and sphingolipid synthesis^36^ and oxysterol sulphate levels.^37^ Given the lack of gross structural neuroanatomical differences in individuals carrying XLI‐associated genetic variants, altered lipid composition of the brain, resulting in comparatively subtle effects (e.g., on cell membrane structure), could explain the observed memory effects.^38^, ^39^

Extension of our preliminary neuroimaging findings in an experimental sample of twice the size, indicated that of the three basal ganglia regions previously reported to be smaller in deletion carriers, only the right globus pallidus finding remained nominally significant. Disruption to globus pallidus‐subthalamic nucleus connectivity has been implicated in hyperactivity, distractibility, over‐rigidity and motor response inhibition^40^ and lower globus pallidus size, and asymmetry, has been associated with idiopathic neurodevelopmental disorders in recent large‐scale meta‐analyses^41^, ^42^; hence, altered right globus pallidus structure/function could partially explain the elevated likelihood of neurodevelopmental conditions in individuals with XLI.

## 5 CONCLUSION

Genetic deletions at Xp22.31 are associated with adverse effects on mood, and reduced performance (and perception of ability) with respect to aspects of memory, but not with large changes in memory‐associated neuroanatomy; these genetic variants may also influence globus pallidus development/function and subsequently neurodevelopmental traits and disorder vulnerability. Importantly, our results suggest that the use of STS inhibitors as memory enhancers in humans, an idea suggested by animal work, may actually have deleterious effects. Future cognitive and neurobiological studies in deletion carriers, and in model systems, should clarify the biological basis of the observed mood and memory findings.

## CONFLICT OF INTEREST STATEMENT

The authors declare no conflict of interest.

## Supporting information


**Table S1:** Volumes of subcortical memory‐associated (sub)regions.
**Table S2:** Global and regional cortical measures.
**Table S3:** Measures of white matter tract integrity.
**Table S4:** Volumes of basal ganglia subregions previously suggested to be sensitive to Xp22.31 deletion.

**Data S1:** Supporting Information.

## Data Availability

Online survey and TestMyBrain data are available within this manuscript or within the Information files. Neuroimaging and genetic data are available upon application to the UK Biobank resource (https://www.ukbiobank.ac.uk/).
